# A rare case report of pyodermatitis vegetans linked with inflammatory bowel disease in juvenile population

**DOI:** 10.1186/s13256-026-06136-2

**Published:** 2026-05-21

**Authors:** Pakeezah Tabasum, Waleed Ahmad, F. N. U. Simran, Mir Raza Ali, Yasmeen Sufi, Leina Elomeiri

**Affiliations:** 1 Peoples University of Medical and Health Sciences for Women, Nawabshah, Pakistan; 2 Lahore Medical and Dental College, Lahore, Pakistan; 3 Bahria University Health Sciences Campus, Karachi, Pakistan; 4 Jinnah Postgraduate Medical Centre, Karachi, Pakistan; 5 Karachi Medical and Dental College, Karachi, Pakistan; 6 University of Khartoum, Khartoum, Sudan

**Keywords:** Pyodermatitis vegetans, Skin lesion, Inflammatory bowel disease, Juvenile population

## Abstract

Pyodermatitis vegetans (PDV) is a rare, chronic inflammatory dermatosis associated with inflammatory bowel disease (IBD), particularly ulcerative colitis. Though PDV has been documented globally, it remains rare in pediatric populations and under-reported in Pakistan. This case highlights an adolescent male with suspected IBD presenting with extensive cutaneous manifestations consistent with PDV. A 17-year-old male presented with a 10-year history of recurrent vegetative skin lesions, which initially started behind the left ear and then involved the face, axillae, back, chest, buttocks, gluteal cleft, and genitals. He also reported red, itchy, ring-like lesions on the limbs with systemic complaints, including nausea, anorexia, constipation, painful defecation, and fresh rectal bleeding. Physical examination revealed multiple verrucous plaques, nodular eruptions, crusted lesions, and acne scars. Blood workup showed microcytic hypochromic anemia and elevated serum IgE (1375 IU/mL). Histopathological examination of the skin biopsy shows features diagnostic of PDV, including hyperkeratosis, parakeratosis, follicular plugging, elongation of rete ridges, and dermal fibrosis with lymphocytic infiltration. Considering the clinical features and laboratory findings, the final diagnosis made is pyodermatitis vegetans. This study emphasizes how crucial it is to treat PDV as an uncommon extraintestinal sign of IBD in teenagers who exhibit persistent vegetative skin lesions. Accurate diagnosis, mimicking exclusion, and prompt therapy intervention to address underlying systemic connections all depend on multidisciplinary collaboration and teamwork.

## Introduction

Pyodermatitis vegetans is a rare, chronic, and relapsing inflammatory dermatitis, often presenting with exudative, vegetative plaques that may affect the scalp, face, and intertriginous areas. In addition, crowded pustules and erosions characterize PSV on mucosal surfaces, often displaying a “snail track” appearance. Pustules mainly affect the oral mucosa and intertriginous areas, such as the axillae, groin, and perinatal regions [1, 2]. Histologically, it is characterized by eosinophilic spongiosis, pseudoepitheliomatous hyperplasia, subcorneal neutrophilic microabscesses, and eosinophil infiltration [3].

PDV is frequently associated with systemic conditions, most notably inflammatory bowel diseases (IBD), such as ulcerative colitis and Crohn’s disease, suggesting an immunological basis to its pathogenesis. Pyodermatitis vegetans (PDV) and pyostomatitis vegetans (PSV) are clinical manifestations of IBD and share similar histopathological findings, including epidermal hyperplasia, localized acantholysis, and a dense mixed inflammatory infiltration with intraepithelial and subepithelial eosinophilic microabscesses [2]. Due to its overlapping clinical and histological features with other dermatological entities, such as pemphigus vegetans, it poses a significant diagnostic challenge. Both conditions feature vegetative, pustular, and eroded skin or mucosal lesions, with PV also showing acantholysis, papillomatosis, neutrophil and lymphocyte infiltration, and a dense eosinophilic dermal infiltrate [4, 5]. Its rarity and often delayed diagnosis can lead to prolonged morbidity. Early recognition, supported by clinicopathologic correlation and exclusion of mimickers, is crucial for optimal management. We present a rare and diagnostically challenging case of pyodermatitis vegetans in a patient with underlying inflammatory bowel disease, highlighting the need for awareness of atypical presentation and the importance of a multidisciplinary diagnostic approach.

## Case presentation

A 17-year-old male patient presented to the Dermatology Department at Jinnah Postgraduate Medical Centre (JPMC), Karachi, with a chronic history of progressively worsening skin lesions. According to the patient, the dermatological symptoms had persisted for the past 10 years and were characterized by recurrent vegetative plaques. Initially, the condition began as papular eruptions located behind the left ear lobule and was associated with serous (watery) discharge. Over time, these lesions gradually progressed in severity and distribution, evolving into ulcerated, crusted plaques. The cutaneous involvement eventually extended to multiple body regions, including the face, chest, bilateral axillae, upper back, buttocks, gluteal cleft, and genitals. The patient also reported the development of red, itchy, ring-like lesions on both upper and lower limbs, consistent with tinea corporis-like morphology.

In addition to skin complaints, the patient experienced several systemic symptoms. These included decreased appetite, disturbed sleep, and gastrointestinal disturbances, notably constipation and painful defecation. He reported fresh rectal bleeding during bowel movements and altered bowel habits; however, the bleeding was not mixed with feces, suggesting a possible mucosal source. Importantly, he denied any urinary symptoms, joint pain, significant weight loss, vomiting, dyspnea, or chest discomfort. There was also no history of preceding trauma to the affected skin areas, which helped narrow down the potential etiologies.

A review of the patient’s systemic history revealed repeated hospital admissions over the years due to similar skin-related complaints. His past medical history did not include chronic conditions, such as tuberculosis, asthma, or diabetes mellitus. The patient had undergone multiple surgical procedures for lipoma excision in the past. He had a blood transfusion approximately 1 month prior to the current admission.

A general physical examination revealed an adolescent with an average body build who appeared clinically pale, raising concern for anemia. There were no signs of digital clubbing, cyanosis, or peripheral edema. Lymphadenopathy was not observed on palpation. Vital signs were within normal limits. The patient was febrile at the presentation. Systemic examination did not reveal any major abnormalities. Per rectal examination, it confirmed the patient’s complaint of painful defecation with fresh blood, again noting that the blood was not mixed with the stool.

The cutaneous examination was particularly striking and confirmed the chronic, relapsing nature of the disease. Multiple verrucous, non-tender plaques were observed across several body sites, including the cheeks, chest, axillae, upper back, buttocks, and gluteal cleft. The buttock lesions were notably large, irregular in shape, crusted, and appeared chronically inflamed. The upper chest demonstrated nodular eruptions, while the back displayed atrophic scars and healed papular lesions. A crusted lesion was noted over the chin, and pock-like scarring was evident on both cheeks. The face and back were marked by cystic acne scars. In addition, the arms and legs had well-defined, red, ring-shaped lesions suggestive of superficial fungal involvement or inflammatory dermatosis.

Laboratory investigations were conducted to further evaluate the underlying systemic status and support the diagnosis. Hematological analysis revealed microcytic hypochromic anemia and reduced mean corpuscular volume. Lymphocyte percentage revealed mild lymphopenia. The detailed laboratory investigations are reported in Table 1. Biochemical investigations showed a serum albumin level of 3.4 g/dL, which is slightly low and could suggest chronic illness or malnutrition. Of particular note, serum IgE was markedly elevated at 1375 IU/mL, supporting a possible allergic or atopic basis for the skin pathology. The patient’s serum ferritin level was elevated at 248 ng/mL, consistent with an ongoing inflammatory process.

**Table 1:** Laboratory investigations at different periods from the day of admission to last follow-up

Labs	Day 1	Day 8	Day 13	Day 26	Day 31	Day 36	Day 50	Day 56	Day 61	Day 70
Hb (g/dl)	8.8	8.7	9.3	9.3	9.3	9.0	8.3	8.7	10.1	9.4
HCT (%)	31.7	31.3	33.9	32.1	32	29.9	27.1	29.2	32.2	32.6
MCV (fl)	68.0	68.6	70.3	67.9	67.9	69.4	60	66	68.9	69.7
MCH (pg)	18.8	19.1	19.2	19.6	19.3	20.9	18.7	19.6	21.5	20.1
MCHC (g/dl)	27.6	27.8	27.4	28.9	28.4	30.1	30.8	29.7	31.4	28.8
RBC (× 10^12^/L)	4.66	4.56	4.83	4.73	4.8	4.3	4.46	4.42	4.68	4.68
PLT (× 10^9^/L)	376	513	430	398	521	497	485	181	490	380
WBC (× 10^9^/L)	16.1	15	14.1	11.3	13	13.5	14.1	16.4	15.2	8.31
Na (mmol/L)	136	139	137	136	138	138	140	NA	NA	NA
K (mmol/L)	4.6	2.9	4.0	3.4	3.68	4.01	4	NA	NA	NA
Cl (mmol/L)	104	109	102	102	101	101	103	NA	NA	NA
T.Bil (mg/dl)	0.25	0.30	0.30	0.30	0.34	0.44	0.67	NA	NA	NA
D.Bil (mg/dl)	0.10	0.12	0.10	0.12	NA	NA	NA	NA	NA	NA
ALT (U/L)	10	13	18	16	11	11	12	NA	NA	NA
GGT (U/L)	23	21	22	26	23	22	23	NA	NA	NA
ALP (U/L)	90	89	92	89	62	92	94	NA	NA	NA
Urea (mg/dl)	52	16	14	22	24	16	09	NA	NA	NA
Creatinine (mg/dl)	0.65	0.66	0.98	0.63	0.4	0.73	0.57	NA	NA	NA

*Hb* Hemoglobin, *HCT* Hematocrit, *MCV* Mean Corpuscular Volume, *MCH* Mean Corpuscular Hemoglobin, *MCHC* Mean Corpuscular Hemoglobin Conc., *RBC* Red Blood Cells, *PLT* Platelets, *WBC* White Blood Cells, *Na* Sodium, *K* Potassium, *Cl* Chlorine, *T.Bil* Total Bilirubin, *D.Bil* Direct Bilirubin, *ALT* Alanine Aminotransferase, *GGT* Gamma-Glutamyl Transferase, *ALP* Alkaline Phosphatase

Urinalysis findings included mild leukocyturia. Pus cultures obtained from skin lesions were negative, indicating no secondary bacterial infection.

**Fig. 1:**
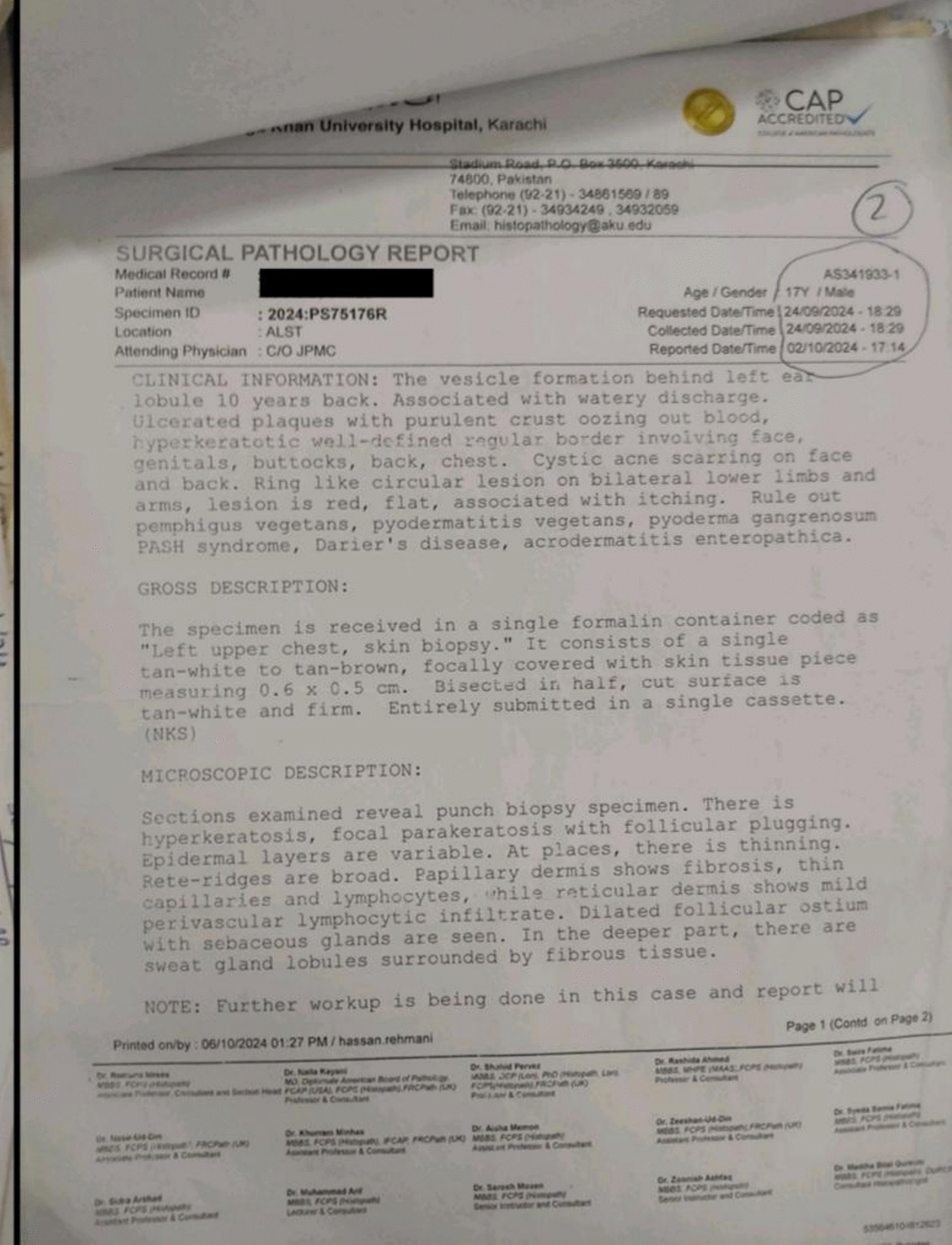
Pathology report of juvenile patient suggestive of pyodermatitis vegetans

Histopathological evaluation of the skin biopsy provided definitive diagnostic insight. The biopsy demonstrated epidermal hyperkeratosis, parakeratosis, follicular plugging, broadening of rete ridges, and focal epidermal thinning, as shown in Fig. 1. There was also evidence of dermal fibrosis and a lymphocytic infiltrate, without features, such as acantholysis or eosinophilic microabscesses. These findings were consistent with a diagnosis of pyodermatitis vegetans. The history of altered bowel habits is slowly suggestive of inflammatory bowel disease.

A thorough differential diagnosis was considered. The primary working diagnosis was pyodermatitis vegetans, supported by the chronic vegetative plaques, elevated serum IgE, and confirmatory biopsy results. Pemphigus vegetans was considered as a close differential but was ruled out due to the absence of acantholysis and eosinophilic abscesses on histology. Pyoderma gangrenosum was also excluded based on the absence of pathergy and typical ulcerative margins. The final diagnosis was established as pyodermatitis vegetans, based on the collective clinical, laboratory, and histopathological findings.

Management was initiated using a combination of systemic and topical therapies. A single dose of azithromycin 250 mg and oxazolidinone 600 mg BD for 14 days was prescribed as an oral antibiotic to cover potential bacterial contributors. Topical therapy consisted of gentamicin cream 2 times daily for a week for local antimicrobial action, along with a mixture of calamine lotion containing resorcinol and 3% sulfur to alleviate itching and inflammation. Additional medications included Medistin 10 mg single dose for 1 month, ATCAM (a nonsteroidal anti-inflammatory drug), and oral corticosteroids in the form of Deltacortil 10 mg for 14 days for immunosuppressive effect.

The patient was admitted under dermatology care for close monitoring and further evaluation. Given the biopsy-confirmed diagnosis and the presence of gastrointestinal bleeding, there was a high index of suspicion for a possible underlying inflammatory bowel disease. Plans were made to investigate this further with stool analysis and potentially endoscopic examination. Supportive management, including nutritional supplementation and correction of anemia, was also initiated to address the patient’s overall health and enhance wound healing.

On 15-day follow-up, the patient reports significant regression in the lesion with decreased erythema, induration, and exudation. No new lesions appeared during the treatment course. No adverse effect has been reported from antibiotics or corticosteroids. Routine labs are within normal range at follow-up. Moreover, the patient was prescribed antihistamine medicine to complete the 1-month course. Maintain good skin hygiene and avoid trauma to affected sites. Advise dermatology follow-up in 2–4 weeks.

## Discussion

Pyodermatitis vegetans (PDV) is a chronic, rare, inflammatory dermatosis proposed as potentially being associated with inflammatory bowel disease (IBD) and with ulcerative colitis (UC) [6]. Initially reported by Hallopeau in the year 1898, PDV presents with exudative, verrucous plaques, usually on intertriginous and periorificial regions, and features of histopathologic changes, such as epidermal hyperplasia with inflammatory infiltrates [7].

The clinical manifestation of this patient, where the patient presented with chronic relapsing verrucous plaques on multiple intertriginous and mucosal surfaces, is consistent with the reported cases of PDV [8]. Histopathologically, review revealed hyperkeratosis, parakeratosis, follicular plugging, elongation of rete ridges, fibrosis of the dermis, and lymphocytic infiltrate with no acantholysis or eosinophilic microabscesses. These results favor PDV to pemphigus vegetans (PV), which, though clinically similar, is characterized by histological presence of suprabasal acantholysis and eosinophilic abscesses [9]. The correlation between PDV and IBD has indicated that more than two-thirds of all cases of PDV resulted in patients who had underlying UC and, less frequently, Crohn's disease [5]. Interestingly, PDV can precede, coincide with, and follow bowel manifestations of IBD, and skin findings may even initiate the assessment and diagnosis of occult IBD [8]. The specific pathogenesis is incompletely appreciated and thought to be associated with immune malregulation, with the increased presence of pro-inflammatory cytokines, particularly interleukin-8, interleukin-17, and tumor necrosis factor-alpha, resulting in neutrophilic dermatoses and injury to mucous membranes [10].

Early recognition of pyodermatitis is clinically important, because it is a rare inflammatory dermatosis that can mimic infectious, autoimmune, or neoplastic conditions, often leading to misdiagnosis and inappropriate treatment [11]. PDV is strongly associated with underlying inflammatory bowel disease, particularly ulcerative colitis and Crohn’s disease, and in some cases, cutaneous manifestation may precede or parallel intestinal disease activity [6]. Failure to identify PDV promptly may delay the diagnosis or recognition of active bowel disease, resulting in prolonged morbidity [12]. For dermatologists, early diagnosis helps avoid unnecessary antimicrobial therapies and invasive procedures, while for gastroenterologists, it serves as an important cutaneous marker of systemic inflammation that may warrant further gastrointestinal evaluation or adjustment of therapy [3, 5]. Therefore, timely recognition of PDV facilitates appropriate multidisciplinary management and improves overall patient outcomes.

Data on long-term outcomes of PDV in pediatric patients remain limited due to its rarity. However, available reports suggest that prognosis largely depends on the control of underlying inflammatory bowel disease, with cutaneous lesions often improving following adequate systemic therapy. Long-term follow-up is recommended to monitor for recurrence and disease activity [13].

This case report is limited by the absence of clinical imaging, including gross and histopathological photographs, and limits the visual documentation of key findings. Although the diagnosis was supported by a detailed report, the lack of imaging may reduce the comprehensiveness and reproducibility of the case for readers. Moreover, the case was documented retrospectively after clinical resolution, with restricted ability to capture real-time clinical progression, lesions, morphology, and treatment response through serial documentation. This may introduce recall bias and limit detailed temporal correlation. Furthermore, the availability of complete visual records may affect external validation and comparison with previously reported cases, particularly in conditions, where morphological characteristics play a significant diagnostic role. Despite these limitations, the case provides valuable clinical insights based on documented findings and a detailed report that contributes to the existing literature on this rare condition.

## Conclusion

This case illustrates the necessity to note the possibility of PDV as a suspected cutaneous unit of underlying systemic illness. It also emphasizes the need for early identification and treatment with a multidisciplinary strategy encompassing pathology, gastrointestinal, and dermatology. Reporting such occurrences helps increase clinical awareness and promotes more research into the connection between PDV and IBD in the juvenile population, especially in areas like Pakistan, where such correlations are infrequently documented.

## Data Availability

The data that support the findings of this study are available on request from the corresponding author. The data are not publicly available due to privacy or ethical restrictions.
